# Do evidence summaries increase policy-makers’ use of evidence from systematic reviews: A systematic review protocol

**DOI:** 10.1186/s13643-015-0116-1

**Published:** 2015-09-28

**Authors:** Jennifer Petkovic, Vivian Welch, Peter Tugwell

**Affiliations:** University of Split School of Medicine, Split, Croatia; Bruyère Research Institute, University of Ottawa, Ottawa, Canada; Public Health and Preventive Medicine, School of Epidemiology, University of Ottawa, Ottawa, Canada; Department of Medicine, Faculty of Medicine, University of Ottawa, Ottawa, Canada; Clinical Epidemiology Program, Ottawa Hospital Research Institute, Ottawa, Canada; Department of Epidemiology and Community Medicine, Faculty of Medicine, University of Ottawa, Ottawa, Canada

**Keywords:** Systematic reviews

## Abstract

**Background:**

Systematic reviews are important for decision-makers. They offer many potential benefits but are often written in technical language, are too long, and do not contain contextual details which makes them hard to use for decision-making. There are many organizations that develop and disseminate derivative products, such as evidence summaries, from systematic reviews for different populations or subsets of decision-makers. This systematic review will assess the effectiveness of systematic review summaries on increasing policymakers’ use of systematic review evidence and to identify the components or features of these summaries that are most effective.

**Methods/design:**

We will include studies of policy-makers at all levels as well as health-system managers. We will include studies examining any type of “evidence summary,” “policy brief,” or other products derived from systematic reviews that present evidence in a summarized form. The primary outcomes are the following: (1) use of systematic review summaries decision-making (e.g., self-reported use of the evidence in policy-making, decision-making) and (2) policy-maker understanding, knowledge, and/or beliefs (e.g., changes in knowledge scores about the topic included in the summary). We will conduct a systematic review of randomized controlled trials (RCTs), non-randomized controlled trials (NRCTs), controlled before-after studies (CBA), and interrupted time series (ITS) studies.

**Discussion:**

The results of this review will inform the development of future systematic review summaries to ensure that systematic review evidence is accessible to and used by policy-makers making health-related decisions.

**Electronic supplementary material:**

The online version of this article (doi:10.1186/s13643-015-0116-1) contains supplementary material, which is available to authorized users.

## Background

Systematic reviews are becoming increasingly important for policy-makers making decisions [1–3]. Systematic reviews offer many potential benefits to policy-makers, including identifying interventions that are effective (or not effective), are considered to have lower risk of bias than other studies, and offer more confidence in results than single studies [2]. However, most systematic reviews are written using technical language, are too long, and do not describe contextual information important for policy-makers and other users making decisions about how to use the evidence [4].

Policy-makers include health ministers and their political staff, civil servants, and health-system stakeholders (i.e., civil society groups, patient groups, professional associations, non-governmental organizations, donors, international agencies [5].

Within the Cochrane Collaboration, the Evidence Aid Project was developed in response to the 2004 Indian Ocean Tsunami as a means of providing decision-makers and health practitioners “on the ground” with summaries of the best available evidence needed to respond to emergencies and natural disasters [6]. A needs assessment conducted by Evidence Aid staff found that systematic review summaries could improve understanding of users (i.e., NGOs, health care providers) so that they can make decisions on the applicability of the findings to their local setting [6]. These user-friendly formats highlight the policy-relevant information and allow policy-makers to quickly scan the document for relevance [2, 7].

In addition to Evidence Aid, there are many organizations that develop and disseminate evidence summaries for different populations or subsets of decision-makers. For example, SUPPORT Summaries were developed for policy-makers in low- and middle-income countries making decisions about maternal and child health programs and interventions (http://www.supportsummaries.org/support-summaries). Health Systems Evidence provides policy briefs for policy-makers making health-systems decisions (www.healthsystemsevidence.org/). Communicate to vaccinate (COMMVAC) is creating user-friendly summaries to translate evidence on vaccination communication for policymakers and the community in LMICs (http://www.commvac.com). Rx for change is a searchable database for evidence about intervention strategies to alter behaviors of health technology prescribing, practice, and use (www.cadth.ca/resources/rx-for-change). In fact, Lavis et al. identified 16 organizations involved in the production of summaries for policymakers in low- and middle-income countries [8].

These summaries may be called evidence summaries, policy briefs, briefing papers, briefing notes, evidence briefs, abstracts, summary of findings, and plain language summaries [8]. They consist of summarized evidence from systematic reviews intended to assist policy-makers in understanding the systematic review evidence and using it in their decision-making. These interventions may include structured summaries (e.g., SUPPORT summaries, Evidence Aid), policy briefs which are based on systematic reviews (e.g., Health Systems Evidence), and plain language summaries, structured abstracts, and Summary of Findings tables (e.g., Cochrane reviews). These may be provided in print or web-based formats and are aimed at policy-makers, and other decision-makers making decisions about health. The summaries may include information about the context in which the studies were conducted, the applicability of the results (e.g., SUPPORT Summaries comment on the relevance of the findings for disadvantaged communities), as well as the findings, methods, and conclusions.

Previously conducted systematic reviews have looked at interventions to increase the use of systematic reviews among decision-makers. For example, Murthy et al. conducted a systematic review examining the effectiveness of interventions for improving the use of systematic reviews in decision-making by health-system managers, policy-makers, and clinicians [9]. Eight studies were included and the authors concluded that information provided as a single, clear message may improve evidence-based practice, but increasing awareness and knowledge of systematic review evidence might require a multi-faceted intervention. Similarly, Perrier et al. conducted a systematic review of interventions encouraging the use of systematic reviews by health policymakers and managers [10]. Four studies were included in the systematic review and the authors concluded that future research should identify how systematic reviews are accessed and the formats used to present the information. Finally, a review by Wallace et al. found that the facilitators to increase systematic review use by policymakers included description of benefits as well as harms and costs, and using a 1:3:25 staged approach to evidence summaries [11]. However, none of these reviews were focused on summaries created from systematic reviews.

Therefore, this review aims to assess the effectiveness of systematic review summaries on increasing policymakers’ use of systematic review evidence and to identify the components or features of these summaries that are most effective.

## Objectives

The objectives of this review are to (1) assess the effectiveness of evidence summaries on policy-makers’ use of the evidence and (2) identify the most effective components of the summaries for increasing policy-makers’ use of the evidence.

## Methods

### Types of studies

We will include randomized controlled trials (RCTs), non-randomized controlled trials (NRCTs), controlled before-after studies (CBA), and interrupted time series (ITS) studies.

### Types of participants

We will include studies which include health policy-makers at all levels (including civil society organization staff, non-governmental organization staff, local government staff, federal government staff) and health system managers making decisions on behalf of a large jurisdiction or organization (6). We will not include studies related to decision-making for an individual person or patient.

### Types of interventions

We will include studies examining any type of “friendly front end,” “evidence summary,” or “policy brief” or other product derived from systematic reviews or guidelines based on systematic reviews that presents evidence in a summarized form to policy-makers and health system managers. Interventions must include a summary of a systematic review and be actively “pushed” to target users. For example, a potentially included study used an intervention that evaluated the effectiveness of friendly front ends by assessing changes in policy-maker beliefs [12]. An example of a study that would be excluded assessed the views of policymakers on how systematic reviews can be promoted within a low- and middle-income country [13].

We will exclude studies in which evidence summaries are one component of a multi-component intervention.

### Comparison

We will include any comparisons including active comparators (e.g., other summary formats) or no intervention.

### Types of outcome measures

#### Primary Outcomes

The primary outcomes are the following:

1. Use of systematic review derivative product in decision-making (e.g., self-reported use of the evidence in policy-making and decision-making as well as self-reported access of research, appraisal of research, or commissioning of further research within the decision-making process [14]. We will include instrumental use of research in decision-making (e.g., direct use of research) as well as conceptual use (e.g., using research to gain an understanding of a problem or intervention) and symbolic use (e.g., using research to confirm a policy/program already implemented) [15].
2. Understanding, knowledge, and/or beliefs (e.g., changes in knowledge scores about the topic included in the summary).

#### Secondary Outcomes

We will also include studies that report on any of the following outcomes:

- Perceived relevance of systematic review summaries
- Perceived credibility of the summaries
- Perceived usefulness and usability of systematic review summaries
  - Perceptions and attitudes regarding the specific components of the summaries and their usefulness
- Understandability of summaries
- Desirability of summaries (e.g., layout, selection of images, etc) [4]

We recognize that some studies may use different terms to describe these outcomes. For example, the term “satisfaction” maybe used as an umbrella term to capture relevance, usability, and desirability. These outcomes will be assessed by the team and categorized according to the above list.

This systematic review has not been registered with PROSPERO since there we are not assessing health outcomes.

### Search methods for identification of studies

An information specialist will help develop the search strategy using the PRESS Guideline [16]. We will build on the search strategy used by Perrier et al. and Murthy et al. in their systematic reviews of interventions to encourage the use of systematic reviews by health managers and policy-makers [9, 10].

### Electronic searches

The search conducted by Perrier et al. identified 11,297 records (after removing duplicates) and included four papers reporting two studies. We will expand this search by including additional databases, as suggested by John Eyres, of the International Initiative for Impact Evaluation (3ie) and the Campbell International Development Review Group. These include databases such as Global Health (CABI), Global Health Library (from WHO), Popline, Africa-wide, Public Affairs Information Service, Worldwide Political Science Abstracts, Web of Science, and DfiD (Research for Development Database) (see Additional file 1).

### Searching other resources

We will search websites of research groups and organizations producing evidence summaries to identify unpublished studies evaluating the effectiveness of the systematic review derivatives in increasing policy-makers’ understanding (e.g., Health Systems Evidence, the Canadian Agencies for Health Technology Assessment, SUPPORT Summaries).

We will check reference lists of relevant studies to identify additional studies. We will contact researchers to identify ongoing and completed/published work. We will report the results of the search using the PRISMA flow diagram.

### Data collection and analysis

#### Selection of studies

Two reviewers will independently screen titles and abstracts to identify relevant studies meeting the pre-specified inclusion criteria. The full text of potentially included studies will be screened independently by two authors. Data extraction and quality assessment will be conducted independently and in duplicate. We will use software Covidence (https://www.covidence.org/) for screening of studies. All completed studies will be included if they meet the inclusion criteria listed above.

### Data extraction and management

The data extraction form will be pre-tested and will include factors related to the population, intervention, comparison, and outcomes. The data will be extracted independently in duplicate by two reviewers using a structured Excel sheet and will be piloted on ten articles. Disagreements on extractions will be resolved by discussion and with a third member of the research team when necessary Evidence summaries, like systematic reviews, that seek to inform decisions in a neutral way should not contain recommendations. Therefore, summaries that provide recommendations will be assessed separately from those without recommendations since these may affect the user experience [17].

Data will be extracted for the following:

- Country
- Setting
- Study design
- Participants
  - Type of policy or decision-makers
  - Country
  - Age
  - Gender
- Intervention
  - Type of evidence summary
  - Format of evidence summary
  - Description of evidence summary components (e.g., descriptions of easy-to-skim formatting, graded entry, use of tables/figures) [18]
  - Mode of delivery
  - Topic of evidence summary
  - Recommendation of evidence summary
- Outcomes
  - Policy/decision-makers’ self-reported use of summaries in decision-making
  - Policy/decision-makers’ knowledge of the summary content and the measurement used
  - Policy/decision-makers’ understanding and measurement used
  - Perceived relevance of the summaries and measurement used
  - Perceived credibility of the summaries and measurement used
  - Perceived usefulness and usability of the summaries and measurement used
  - Perceived understandability of the summaries and measurement used
  - Perceived desirability of the summaries and measurement used
- Process indicators
  - How the systematic review was selected for summary (e.g., based on topic, quality criteria)
  - How the evidence summary was developed (e.g., iterative process)
  - Involvement of stakeholders in evidence summary development—which stakeholders, description of involvement

### Assessment of risk of bias in included studies

The methodological quality will be specifically examined using the risk of bias tools from the Cochrane Handbook for randomized trials and the Effective Practice and Organization of Care (EPOC) Review Group criteria for interrupted time series and controlled before-after studies [19, 20] and A Cochrane Risk Of Bias Assessment Tool: for Non-Randomized Studies of Interventions (ACROBAT-NRSI) [21].

### Measures of treatment effect

Effect estimates and confidence intervals for individual studies will be calculated (where possible) irrespective of whether a pooled effect estimate is calculated. When it is possible to combine studies, dichotomous outcomes will be reported as relative risks. Continuous outcomes will be reported as weighted mean differences. If an outcome has been reported in different scales (e.g., understanding), and we consider the scales to measure a similar construct, standardized mean differences will be used to summarize the data. When it is not possible to combine the data, we will present the results for each study separately.

### Unit of analysis issues

When possible, any studies with cluster allocation (e.g., cluster-randomized trials, cluster-allocated controlled before and after studies, and interrupted time series) analyses with errors in the unit of analysis will be adjusted using the variance inflation factor, as described in the Cochrane Handbook, if the necessary data can be obtained from the study authors. We will obtain ICC from other similar studies with similar outcomes if the ICC is not published (e.g., by checking the Aberdeen website of ICCs, http://www.abdn.ac.uk/hsru/research/delivery/behaviour/methodological-research/ or the Campbell Collaboration website of ICCs for education). Sensitivity analyses will be used to assess the effects of incorporating these corrected analyses in our analysis.

### Dealing with missing data

We will attempt to contact the contact author of the studies by email for any missing data.

### Assessment of heterogeneity

If meta-analysis is possible, we will explore heterogeneity using forest plots and the *I*^2^ statistic according to guidance of the Cochrane Handbook for Systematic Reviews of Interventions [19].

### Assessment of reporting biases

If more than ten studies are included, we will use funnel plots to explore publication bias.

### Data synthesis

Where appropriate, results will be synthesized using meta-analysis. We will present the relative risks using random effects models for dichotomous outcomes and standardized mean differences for continuous outcomes. When studies have reported the same outcome using different scales, we will use standardized mean differences. Non-randomized studies will be meta-analyzed separately from RCTs. When results cannot be pooled, we will present a narrative summary of the results.

We will analyze the results of qualitative data from included studies, when possible, to understand the perceptions and attitudes regarding the components of the summaries that were considered the most useful.

### Assessing the methodological quality

We will use the Grading of Recommendations Assessment, Development and Evaluation (GRADE) approach to assess the quality of evidence for the outcomes reported in this review [22].

### Subgroup analysis and investigation of heterogeneity

Heterogeneity will be explored, if possible, by conducting meta-regression to assess the role of mediating factors, including the following:

- Target audience of summary (e.g., focused on specific local context, generic summary)
- Type of decision maker (e.g., federal policy-maker versus hospital administrator)
- Components of friendly front end (e.g., bulleted list, text, summary of findings table, causal chain)

### Sensitivity analysis

The impact of including studies assessed as high risk of bias or studies in which there were unit of analysis errors that could not be reanalyzed will be considered in sensitivity analysis.

### Applicability

We will assess applicability of the findings of the review to specific settings of relevance to end-users. We will use the most up to date methods from the Cochrane Applicability and Recommendations Methods Group which include assessing the directness of the evidence to specified settings of interest using GRADE [23].

## Discussion

This review will summarize the evidence on the use of systematic review summaries in policy-making and policy-makers’ understanding of systematic review evidence, and assess evidence about different components and design features. To the best of our knowledge, this is the first systematic review to assess the use of systematic review summaries in policy-making. The results of this review will inform researchers and systematic review summary developers of the best way to present the evidence to ensure that evidence summaries fulfill their goal of informing policy-makers with the best possible evidence needed to make health-related decisions.

## Additional file

Additional file 1:
**Appendix 1.** (DOC 17 kb)

## Abbreviations

3ie: International Initiative for Impact Evaluation
CABI: Centre for Biosciences and Agriculture International
CBA: controlled before-after studies
CIHR: Canadian Institutes of Health Research
COMMVAC: communicate to vaccinate
DfiD: Department for International Development
GRADE: Grading of Recommendations Assessment, Development and Evaluation
ICC: intracluster correlation coefficient
ITS: interrupted time series
NGO: Non-Governmental Organization
NRCT: non-randomized controlled trial
RCT: randomized controlled trial
WHO: World Health Organization
